# Endoscopic Delivery of Hydrogels: A Novel Strategy for Treating Early-Stage Gastrointestinal Tumors

**DOI:** 10.3390/bioengineering13060681

**Published:** 2026-06-12

**Authors:** Yunbo Jia, Nan Ge

**Affiliations:** 1 Department of Gastroenterology, Shengjing Hospital of China Medical University, Shenyang 110004, China; jiayunbo0211@163.com; 2 Engineering Research Center of Ministry of Education for Minimally Invasive Gastrointestinal Endoscopic Techniques, Shengjing Hospital of China Medical University, Shenyang 110004, China

**Keywords:** hydrogels, gastrointestinal tumors, endoscopic submucosal dissection, chitosan, hyaluronic acid, sodium alginate

## Abstract

This review systematically illustrates the application and research progress of endoscopically delivered hydrogels as a novel strategy in the endoscopic treatment of early-stage gastrointestinal tumors. It focuses on analyzing the unique physicochemical properties, biological functions, and clinical value of hydrogels as submucosal injection materials, and delves into their core roles in achieving sustained mucosal lifting, effective hemostasis, and wound repair during endoscopic submucosal dissection (ESD). Representative hydrogel materials, such as chitosan, hyaluronic acid, and sodium alginate, are driving the evolution of ESD technology from a mere “resection” procedure toward an integrated “lift-resect-repair” therapeutic model, owing to their excellent biocompatibility, injectability, and controllable degradability. Although challenges in clinical translation remain, including long-term safety, precise control of degradation rates, and scalable production, the field is poised for further breakthroughs with the development of smart responsive hydrogels and their deep integration with emerging technologies.

## 1. Introduction

Gastrointestinal malignancies, encompassing esophageal, gastric, and colorectal cancers, constitute a significant global health challenge, accounting for more than 20% of all cancer-related mortalities worldwide [1,2]. The reduction of mortality associated with these malignancies fundamentally depends on early detection, accurate diagnosis, and timely therapeutic intervention. Endoscopy serves a pivotal function in the early screening and identification of these conditions [3]. During endoscopic examination, submucosal tumors (SMTs) are frequently encountered [4]; however, distinguishing their origin and pathological nature remains challenging due to the similar presentation of various SMTs—both benign and malignant—as smooth, protruding lesions [5,6,7,8]. The integration of advanced endoscopic technologies has markedly improved diagnostic precision in diseases of the digestive system and has catalyzed extensive research efforts aimed at refining endoscopic diagnostic methodologies [9,10,11,12,13].

Concurrently with diagnostic advancements, endoscopic technology has progressed from a primarily diagnostic modality to a robust therapeutic platform [14]. Endoscopic submucosal dissection (ESD) has emerged as a minimally invasive, cornerstone technique for the management of early-stage gastrointestinal cancers, demonstrating favorable prognostic outcomes and high survival rates [15,16,17]. ESD facilitates en bloc resection of large lesions exceeding 2 cm, thereby enabling precise pathological assessment and accurate staging [18,19,20]. Consequently, ESD is now recognized as one of the principal treatment approaches for early gastrointestinal tumors [21,22,23]. Despite its advantages, including minimal invasiveness and cost-effectiveness, ESD presents considerable technical challenges [24]. Primarily, the procedure is complex; conventional submucosal injectates such as normal saline (NS) dissipate rapidly, often requiring repeated administration and failing to maintain a stable and sustained submucosal cushion during intricate dissections, which may compromise procedural safety and efficiency. Additionally, the procedure carries risks of complications including bleeding, perforation, and localized inflammation at the resection site. For extensive or irregular mucosal defects, traditional closure techniques, such as metallic clips, demonstrate limited effectiveness. Therefore, the development and identification of more efficacious submucosal injection materials are imperative to mitigate postoperative complications and enhance patient outcomes.

Hydrogels have emerged as a promising strategy to address these limitations. Defined as three-dimensional polymer networks capable of retaining substantial amounts of water, hydrogels are broadly categorized into natural and synthetic biomaterials [25]. Hydrogels based on natural polymers, such as those derived from chitosan (CS) [26,27,28], sodium alginate (SA) [29,30], and hyaluronic acid (HA) [31,32], are particularly favored for their excellent biocompatibility, including low cytotoxicity, negligible immunogenicity, and tunable biodegradation kinetics [33,34]. They play a crucial role in wound healing, bone repair and the treatment of arthritis, etc. The amino and hydroxyl functional groups in them can be chemically modified to enhance mechanical strength and biological activity, thereby expanding their application range [35]. Their long-lasting mucosal elevation ability and promoting wound healing properties are particularly suitable for endoscopic treatment. The hydrogel remains in a fluid state during injection and, after being injected into the submucosal layer, can quickly solidify under physiological conditions (such as temperature, pH, enzymatic action or external light-induced polymerization), making the operation convenient and reducing the need for repeated injections during ESD surgery.

In summary, the introduction of endoscopically deliverable hydrogels is essential in therapeutic endoscopy. This review aims to summarize the development and application of hydrogels in ESD, focusing on their physicochemical properties, underlying mechanisms of action, biocompatibility, and clinical significance. By addressing the key challenges in ESD for early-stage tumors, hydrogels are advancing the field from a simple “resection” approach toward an integrated “lift-resect-repair” therapeutic model.

## 2. Hydrogels for Endoscopic Resection: Classification and Fundamental Properties

Endoscopic resection (ER) is a minimally invasive technique for the local removal of early gastrointestinal tumors without lymph node metastasis [36,37]. Compared to traditional surgery, ER significantly reduces trauma and improves patients’ postoperative quality of life [38]. The primary techniques include polypectomy, endoscopic mucosal resection (EMR), and ESD. EMR has limitations in achieving en bloc resection of large lesions, often resulting in incomplete pathological specimens, residual lesions, and diagnostic challenges. The development of ESD has largely addressed these issues by enabling complete resection of large lesions, facilitating thorough pathological assessment, and reducing the risk of recurrence [16]. Consequently, ESD offers significant advantages, particularly for extensive lesions in the esophagus and stomach. However, despite its benefits, ESD is technically demanding, requires longer procedure times, and carries risks of complications such as perforation, bleeding, esophageal stricture, and persistent inflammation [22]. The ESD procedure typically involves three key steps: first, marking the lesion boundaries; second, injecting fluid into the submucosa to lift the lesion, creating a submucosal fluid cushion and performing a circumferential incision; and finally, completing the submucosal dissection using electrosurgical devices [39].

A critical factor for procedural success is the selection of an appropriate submucosal injection material. An ideal material must effectively separate the mucosal layer from the muscularis propria, providing a stable lesion lift to facilitate rapid and safe dissection [40,41]. Traditional agents, such as NS, diffuse quickly and often require frequent reinjection, which prolongs procedure time and increases the risk of tissue inflammation. This underscores the urgent need for more effective injectates that can maintain lifting for longer durations and reduce complications. According to the American Society for Gastrointestinal Endoscopy, an ideal submucosal injection agent should possess the following characteristics: (1) create an adequate submucosal fluid cushion thickness; (2) maintain the cushion long enough to complete the ESD procedure; (3) preserve specimen integrity for accurate pathological examination; (4) be cost-effective, easily accessible, and storable; (5) be non-toxic and harmless to tissue; and (6) be easy to inject.

**Figure 1:**
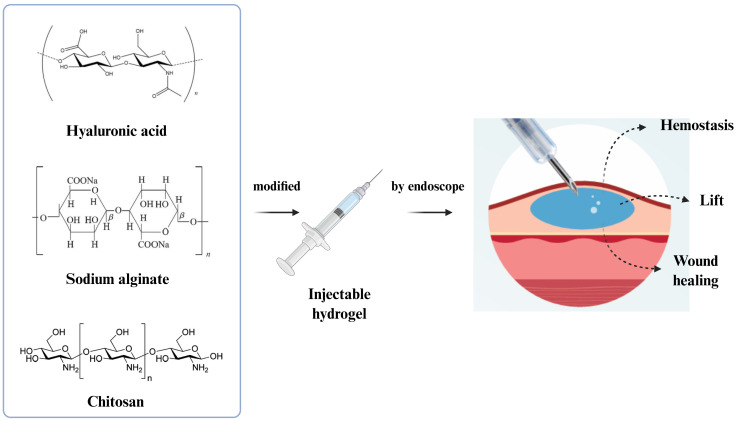
Injectable hydrogels (CS, SA and HA) for ER.

Hydrogel materials, represented by CS, SA, HA, and their derivatives, exhibit essential properties such as biocompatibility, injectability, degradability, and modifiability (Table 1) (Figure 1). These characteristics provide the foundational basis for their wide range of clinical applications [42] (Table 2).

(1) CS: CS is derived from the deacetylation of chitin found in the exoskeletons of crustaceans and arthropods [44]. It exhibits high adhesiveness, hemostatic and antibacterial properties, and low cytotoxicity [43,45]. However, its inherent insolubility in neutral pH aqueous solutions limits its direct biomedical applications. Modified CS derivatives can serve as submucosal injectates. For example, modification of free amino groups with lactose and photoreactive azide groups produces fully water-soluble CS derivatives at neutral pH, which form insoluble hydrogels upon UV irradiation, making them suitable for submucosal injection. The high viscosity of CS makes it difficult to inject through endoscopic needles. To address this, a team studied photocrosslinked CS, a material that can be injected into the submucosal layer of the digestive tract in solution form. Upon exposure to ultraviolet light, photocrosslinked CS transforms into a hydrogel, providing a significant and long-lasting lifting effect [54]. However, reliance on ultraviolet light raises concerns about potential adverse effects on surrounding normal tissues with long-term use. Therefore, temperature-sensitive hydrogels based on CS and β-glycerophosphate (CS/GP), known as thermo-sensitive CS, were developed [55]. These hydrogels remain in solution at low temperatures and transform into a gel at body temperature. Nevertheless, the gelation process of CS/GP is slow. To optimize this, a temperature-sensitive gel system containing CS, β-glycerophosphate, and collagen (CS/GP/Col) was developed, which accelerates gelation, stimulates growth factor secretion, and modulates local inflammatory responses [56]. However, this system exhibits poor bioadhesion and is prone to detachment. To overcome these limitations, hydroxypropyl cellulose (HPC) was added to the CS/GP/Col system, resulting in a novel four-component gel system (CS/GP/HPC/Col) characterized by rapid gelation, enhanced adhesion, and reduced inflammatory response [56].
(2) SA: SA is a natural polysaccharide extracted from brown algae, composed of linear chains of β-D-mannuronic acid (M) and α-L-guluronic acid (G) residues linked by 1,4-glycosidic bonds [46]. Variations in the M/G ratio and block structures result in differences in conformation and biological properties [57,58], allowing modulation of hydrogel mechanical strength and bioactivity through control of molecular weight and distribution. SA promotes platelet aggregation and accelerates fibrin formation. Engineering modifications, such as adjusting SA concentration [59] or calcium ion concentration [60], can enhance its functional properties to regulate mechanical strength and stability. Alginate exhibits favorable mechanical properties by providing spatial support and filling, excellent biocompatibility, low immunogenicity, absence of specific cell recognition sites, and resistance to degradation by mammalian enzymes, demonstrating relative bio-inertness [9,50]. Alginate gel preparation is simple, mild, and non-toxic [49]. Grafting and modifying alginate chains to introduce specific functional groups can expand its applications in drug delivery, bio-coatings, wound healing, and tissue engineering scaffolds [47,48]. SA composites with CS [61] or polyacrylamide [62] can serve as wound dressings with effective hemostatic and pro-healing properties [63]. In a comparative experimental study, ESD was successfully accomplished with 2%, 3% and 4% SA in 10 patients [63]. Studies show that SA solutions at concentrations between 0.6% and 4% are more effective submucosal injectates than NS, with 0.6% considered optimal, providing effective tissue lifting without causing damage [63,64]. Uemura et al. applied 0.6% SA during ESD for gastric and esophageal tumors and found its efficacy [65,66]. In 2024, a single-center, retrospective pilot study shows that the rate of en bloc resection of 0.6% SA group was 97% [67]. Both in vitro and ex vivo studies have confirmed the effectiveness of SA. As a material with great potential, its future clinical utility can be further enhanced by optimizing concentration, formulation, and performance.
(3) HA: HA is an acidic glycosaminoglycan composed of D-glucuronic acid and N-acetylglucosamine, widely found in mammalian connective tissues [51]. Valued for its excellent water retention, ability to promote cell repair, and high biocompatibility, HA is extensively used in fields such as ophthalmology and orthopedic treatments [52]. The safety and efficacy of HA have been demonstrated in both large animal models and human studies. Due to its high viscosity, HA requires dilution prior to injection.

High-concentration HA demonstrates certain elasticity and adhesiveness. A 0.4% HA solution has been identified as a suitable concentration for submucosal injection, making it one of the most widely used alternatives to NS in clinical practice [53]. Besides, a single-center prospective randomized controlled trial and a single-arm multicenter prospective open trial indicate that, compared with NS, 0.13% HA is a better option, as it increases the rate of complete lesion removal and reduces the incidence of complications [68]. It is easy to inject, provides reliable tissue lifting, and enhances resection efficiency. However, the relatively high cost of raw material extraction limits its broader adoption as a submucosal injectate. To reduce costs, HA solutions are often mixed with other substances, such as a mixture of 0.125% HA derived from a 1% 1900 kDa HA preparation combined with 10% glycerol, 5% fructose, and 0.9% NS [69], or a solution consisting of HA, chondroitin sulfate, and poloxamer 407, which was tested in a porcine in vivo model [70]. These mixtures provide long-lasting submucosal cushioning at a lower cost [70].

**Table 2:** Comparison of studies on CS, SA and HA.

Year	Type of Study	Components	Results
2012	Animal experimental study	Photocrosslinkable CS hydrogel	A long-lasting lifting effect [54].
2021	Animal experimental study	CS/GP	It remains in solution at low temperatures and transforms into a gel at body temperature [55].
2024	Animal experimental study	HpHCS-PVP-β-glycerophosphate	It enhances the stability and injectability, and enables rapid gelation even at low concentrations [71].
2011	A comparative experimental study	2%, 3%, 4% SA	ESD was successfully accomplished with SA in 10 patients [63].
2019	A multicenter randomized controlled trial	0.6% SA	During ESD, the efficacy of submucosal injections was 91.7% [66].
2024	A single-center, retrospective pilot study	0.6% SA	The rate of en bloc resection was 97% [67].
2006	Clinical study	A mixture of 0.125% HA derived from 1% 1900 kDa HA with 10% glycerol, 5% fructose, and 0.9% NS.	Endoscopic en bloc resection rate was 94% but histologic en bloc resection rate was 78% [69].
2012	Prospective randomized controlled trial	0.13% HA	The complete resection rate was significantly higher than the NS group [68].
2017	Animal experimental study	HA combined with chondroitin sulfate and poloxamer 407	Reducing cost and long-lasting [70].
2023	A comparative experimental study	A temperature-sensitive hydrogel based on HA and poloxamer 407	A longer duration of elevation compared to NS [72].

## 3. Application Strategies Based on Therapeutic Objectives

### 3.1. For Safe Resection: Submucosal Lifting Hydrogels

Safe and effective submucosal lifting is essential for successful ESD. Traditional fluid cushions are short-lived, which can result in loss of the surgical field and an increased risk of perforation.

Injectable submucosal lifting hydrogels can form a stable, long-lasting physical barrier within the submucosa, effectively separating the mucosal layer from the underlying muscle. Compared to NS, these hydrogels extend the lifting duration from minutes to several hours, providing ample time for precise dissection. Their elastic properties also buffer surgical manipulation, reducing the risk of perforation. Examples include cross-linked HA gels and alginate-gelatin composite gels. A novel composite thermosensitive hydrogel based on high-pH CS(HpHCS)-polyvinylpyrrolidone(PVP)-β-glycerophosphate has been developed. Here, HpHCS improves injectability and enables rapid gelation even at low concentrations; modified PVP significantly enhances the stability of the low-temperature hydrogel precursor solution and the integrity of the HpHCS thermosensitive hydrogel formed via hydrogen bonding at body temperature [71]. This novel composite thermosensitive hydrogel avoids the need for repeated injections during dissection and shows no cytotoxicity after electrocautery, demonstrating its potential for ESD application. In 2023, a temperature-sensitive hydrogel based on HA and poloxamer 407 exhibited high stiffness and viscosity at 37 °C while maintaining good injectability [72]. It was safe in pig mucosa and effectively prolonged mucosal elevation time [72]. Subsequently, a research team prepared oxidized HA and acylhydrazide HA through oxidation and adipic dihydrazide modification, selecting the most optimal hydrogel. This hydrogel demonstrated ideal injectability, gelation time, mechanical strength, and excellent cell compatibility. In a pig ESD surgery model, it significantly increased lift time and height. The surgery could be completed in as little as ten minutes, and the hydrogel adhered to small bleeding points, protecting the wound. These findings indicate that this material has great potential to improve the efficiency of ESD surgeries and to contribute to the development of new biomedical materials [73].

Hydrogels combining alginate with other components have been widely explored for submucosal cushion development. For example, SA combined with calcium lactate to form a calcium alginate gel demonstrated a significantly greater lifting height than NS and glycerol groups 30 min post-injection in an ex vivo gastric model [63]. Alginate can also be formulated into microspheres. One study successfully prepared two types of alginate polymer microparticles (powder and hydrogel) using emulsion/internal gelation technology [74]. These microparticles exhibited good injectability and provided significant lifting in porcine gastric and colonic mucosa.

### 3.2. For Intra- and Post-Operative Safety: Hemostatic and Sealing Hydrogels

Managing acute bleeding during and after ESD is critical [75]. Biologically, an ideal injectable hydrogel should cause minimal tissue damage, aid hemostasis, promote postoperative wound healing [76,77], and prevent fibrosis. Hemostatic hydrogels function by forming a physical barrier to compress bleeding sites and/or by activating coagulation pathways through their intrinsic material properties (e.g., CS [78]) or loaded hemostatic agents (e.g., tranexamic acid). For example, a pH-responsive, self-healing adhesive hydrogel can gelate in the acidic gastric environment, resist peristalsis, and deliver drugs (e.g., ε-aminocaproic acid) to achieve rapid hemostasis and promote healing [79]. CS-based submucosal injectates not only demonstrate excellent lifting capacity but also have potential to promote wound healing and reduce bleeding. SA has the ability to promote platelet aggregation and accelerates fibrin formation, playing a significant role in hemostasis during ESD. Furthermore, a versatile dual-component in situ hydrogel derived from SA and hydroxymethyl CS has been developed [80]. Unlike traditional thermosensitive gels, this hydrogel utilizes “click” chemistry and Schiff base reactions, enabling delayed gelation upon contact with wounds. It features rapid gelation and excellent tissue adhesion. Animal studies have confirmed its rapid hemostatic effect, effective mucosal lifting, wound closure capability, and significant pro-healing action, demonstrating great potential for gastrointestinal ESD applications.

Reliable closure of ESD wounds is essential for preventing delayed bleeding and perforation. Endoscopically sprayable “biological bandages” are delivered as powders or liquids via endoscopy [81]. They rapidly cross-link on the wound surface, forming a tough, elastic protective film. Their advantages include ease of application, complete coverage—especially for large or irregular wounds—and effective isolation of the wound from gastric acid and gastrointestinal contents. A hybrid dry powder based on polyacrylic acid and other components can gel within 5 s upon contact with wound moisture, exhibits strong adhesive strength (>30 kPa), and remains stable in the gastric environment, offering a novel option for post-ESD wound closure [82]. By utilizing in situ crosslinking and shear-thinning/self-repairing properties, injectable HA hydrogels enable precise filling of lesions, provide post-gelation support to surrounding tissues, and enhance targeted delivery of therapeutic agents for effective tissue regeneration.

Recently, researchers have developed a novel multifunctional and clinically applicable hydrogel capable of both sustained intraoperative mucosal elevation (to ensure a clear surgical field) and active postoperative promotion of tissue repair, including anti-inflammatory effects, angiogenesis stimulation, and fibrosis inhibition [83]. This material is designed based on dynamic cross-linking and ionic coordination mechanisms, exhibiting unique properties such as injectability, self-healing ability, and tunable gelation kinetics. The hydrogel system is synthesized through the dynamic cross-linking of SA and carboxymethyl CS, with its mechanical strength and hemostatic properties enhanced by calcium ion coordination. A novel component introduced in this study is the natural antioxidant protein phycocyanin, which not only imparts a blue color for visual contrast during surgery but also confers bioactive immunomodulatory functions to the hydrogel. This hydrogel demonstrates adjustable gelation kinetics, excellent injectability, self-healing capacity, and compatibility with endoscopic delivery systems [83].

### 3.3. Enhanced Function: Hydrogels in Surgical Training

Significant advancements have been made in surgical simulation through the development of tissue-simulating hydrogels specifically engineered for ESD skills training. Notably, a high-fidelity ESD training model has been created using a novel double-network composite hydrogel [84]. This material accurately replicates the complex biomechanical properties of authentic gastric tissue, including elasticity, tensile strength, and mucosal layer resistance. By providing exceptionally realistic haptic feedback and anatomical response, these advanced hydrogel-based models substantially enhance the precision and efficiency of surgical training. They offer a safe, reproducible, and risk-free environment for trainees to master delicate techniques, from submucosal injection to precise dissection [85]. Consequently, this indirect yet powerful contribution translates into significantly improved clinical procedural safety and patient outcomes, effectively bridging the critical gap between theoretical knowledge and hands-on surgical expertise.

## 4. Challenges and Future Perspectives

Endoscopic technology has evolved from a diagnostic tool into a therapeutic platform, enabling the delivery of biological scaffolds. Currently, the most commonly used submucosal injection material in clinical practice is NS. Due to its low cost and high biocompatibility, NS diffuses rapidly and is quickly absorbed by surrounding tissues, which prevents it from maintaining the desired submucosal elevation. Hydrogels have demonstrated superior operational characteristics and therapeutic efficacy compared to NS in both animal and clinical studies. Despite this encouraging progress, existing hydrogel materials still fall short of the ideal criteria for injectates, and their clinical translation faces multiple challenges. Key hurdles include ensuring the long-term in vivo safety of hydrogels, precisely matching their degradation rates with wound healing processes, reducing the costs of scalable production, enhancing mechanical strength and biological activity, and minimizing complications during and after surgery [86,87]. The degradation products of hydrogel biomaterials are generally non-toxic. Chemical modification is often employed to address these issues; however, such complex processes may adversely affect biocompatibility and functionality. Innovative exploration of new modification techniques and combination strategies is crucial to overcoming these challenges [88]. Rigorous validation through systematic animal experiments and multicenter clinical trials is essential for comprehensively assessing the safety, efficacy, and long-term stability of these materials.

In the future, designing hydrogels that can be customized to match individual patients’ wound sizes and shapes will be highly valuable. Furthermore, developing “smart” responsive hydrogels capable of reacting to specific biological signals—such as pH changes at bleeding sites or specific enzymes in the tumor microenvironment—is an important direction. These hydrogels should feature rapid gelation and excellent tissue adhesion. Continuous innovation in endoscopic devices and operational modes, combined with emerging technologies [89,90,91], will further advance technological convergence and development in this field. The criteria established by the American Society for Gastrointestinal Endoscopy regarding cost-effectiveness and ease of accessibility aim to define an ideal hydrogel, which is expected to see widespread use in clinical practice.

## 5. Conclusions

In summary, the endoscopic delivery of multifunctional hydrogels that integrate lifting, hemostasis, and sealing capabilities paves the way for advanced endoscopic therapies. Developing modified injectable hydrogels tailored to specific clinical needs represents a crucial direction for future research. This interdisciplinary approach, combining the strengths of materials science and minimally invasive technology, offers a safer and more effective pathway for treating early-stage gastrointestinal tumors endoscopically. With continued material innovation and technological advancement, this strategy holds great promise for further improving therapeutic outcomes and enhancing the quality of life for patients with early gastrointestinal tumors.

## Figures and Tables

**Table 1:** Comparison of CS, SA and HA.

Hydrogels	Structure	Benefits	Limitations
Chitosan	a copolymer of N-acetylglucosamine and D-glucosamine [43,44]	High adhesiveness; hemostasis; antibacterial properties; low cytotoxicity [43,45].	Limited solubility; high viscosity [43,45].
Sodium alginate	linear chains of β-D-mannuronic acid (M) and α-L-guluronic acid (G) residues linked via 1,4-glycosidic bonds [46]	Inexpensive; easy to get and store; excellent biocompatibility; low immunogenicity; hemostasis [47,48,49,50].	High concentration injection difficulty; optimal concentration yet to be determined [47,49,50].
Hyaluronic acid	an acidic glycosaminoglycan composed of D-glucuronic acid and N-acetylglucosamine [51]	Good biocompatibility; excellent water retention; promoting cell repair [52].	Costly; high viscosity; limited availability [53].

## Data Availability

No new data were created or analyzed in this study. Data sharing is not applicable to this article.

## Abbreviations

The following abbreviations are used in this manuscript:

CS	Chitosan
CS/GP	Chitosan and β-glycerophosphate
CS/GP/Col	Chitosan, β-glycerophosphate, and collagen
EMR	Endoscopic mucosal resection
ER	Endoscopic resection
ESD	Endoscopic submucosal dissection
HA	Hyaluronic Acid
HpHCS	High-pH chitosan
NS	Normal saline
SA	Sodium Alginate
SMTs	Submucosal tumors
